# Characterization and Antibacterial Activity of Phthalides from the Roots of the Medicinal Herb *Levisticum officinale* W.D.J. Koch.

**DOI:** 10.22037/ijpr.2020.112583.13839

**Published:** 2020

**Authors:** Mansour Miran, Hamidreza Monsef Esfahani, Jee Hyung Jung, Atousa Aliahmadi, Danielle Skropeta, Mahdi Abbas-Mohammadi, Samad Nejad Ebrahimi, Mahdi Moridi Farimani

**Affiliations:** a *Department of Pharmacognosy and Biotechnology, School of Pharmacy, Ardabil University of Medical Sciences, Ardabil, Iran. *; b *Department of Pharmacognosy, Faculty of Pharmacy, Tehran University of Medical Sciences, Tehran, Iran. *; c *College of Pharmacy, Pusan National University, Busan, South Korea. *; d *Department of Biology, Medicinal Plants and Drugs Research Institute, Shahid Beheshti University, G.C., Evin, Tehran, Iran. *; e *Molecular Horizons and School of Chemistry & Molecular Bioscience, University of Wollongong, Wollongong, NSW 2500, Australia. *; f *Department of Phytochemistry, Medicinal Plants and Drugs Research Institute, Shahid Beheshti University, G.C., Evin, Tehran, Iran.*

**Keywords:** Levisticum officinale, Phthalide, Structure elucidation, Antibacterial activity

## Abstract

A new phthalide, namely 7-methoxy-3-propylidenephthalide (**1**), along with two known compounds (**2**, **3**) were isolated from the roots of the edible herb *Levisticum officinale *W.D.J. Koch, commonly known as lovage and well known in traditional medicine for its spasmolytic and diuretic effects. The structure of the new compound was established by HRMS and 1D & 2D NMR (^1^H ^1^H COSY, HMQC, and HMBC) spectroscopic analysis. All compounds are reported for the first time from *L. officinale*. Compounds **1-3** were tested against two Gram negative (*Escherichia coli*, *Pseudomonas aeruginosa*) and two Gram positive (*Staphylococcus aureus* and vancomycin-resistant *Enterococcus* [VRE]* faecium*) bacteria strains. Compound **3 **was active against *S. aureus*, *E. coli* and vancomycin-resistant *E. faecium* with MIC values of 16, 64, and 128 μg/mL, respectively.

## Introduction

Phthalides are a class of secondary metabolites with a wide range of pharmacological activities including inhibition of various enzymes, anti-inflammatory, anti-atherosclerosis, blood viscosity reduction, anti-angina, anti-convulsion, and antimicrobial activities (1-4). Phthalides and their derivatives including dihydro, tetrahydro, and dimeric compounds have been found in several species of the *Apiaceae* family (2). 

*Levisticum officinale*, a member of the Apiaceae family, is a rich source of phthalides and their derivatives including (Z)-ligustilide, (Z)-3-butylidenephthalide, (E)-3-butylidenephthalide, 3-butylphthalide, and levistolide A (5, 6). 

*L. officinale* grows widely in the Hezar Mountain of the Kerman Province in Iran. The root of this plant has been used for various biological effects primarily as a diuretic, as a treatment for urinary tract infection and as a spasmolytic agent (6-8).

In order to discover new and potentially bioactive constituent from *L. officinale*, we investigated the ethyl acetate extract of the root. In this study, we report on the isolation, structural identification, and antibacterial activity of three phthalides, including a new one.

## Experimental

*General experimental procedures*

NMR spectra were recorded on a Bruker AVANCE III spectrometer operating at 500 MHz for ^1^H NMR and 125 MHz for ^13^C NMR. UHPLC-MS analyses were performed on a Waters Acquity UPLC system coupled to a Waters Xevo^TM^ quadrupole time-of-flight (QToF) mas spectrometer and equipped with an electrospray source with lockspray interface for accurate mass measurements. Silica gel (70-230 mesh) was used for column chromatography, and precoated silica gel F_254_ (20 × 20 cm) plates for TLC, both supplied by the Merck. C_18_-reversed phase silica gel used for column chromatography, was purchased from Sigma.

*Plant material*

The roots of *L. officinale* were collected in July 2015 from the Hezar Mountain of Kerman Province, Iran. The plant material was identified by Prof. Farideh Attar from Tehran University. A voucher specimen (46553-TUH) has been deposited in the herbarium of the Science Faculty of Tehran University, Iran.

*Extraction and isolation*

Air-dried, powdered roots of *L. officinale* (3 kg) were extracted with *n*-hexane (3 x 9 L) and then ethyl acetate (3 x 9 L) by maceration at room temperature. The ethyl acetate extract was concentrated by rotary evaporation, to afford 100 g of dried extract. This extract was subjected to silica gel column chromatography (230- 400 mesh, 1 kg), with a gradient of n-hexane–EtOAc and then EtOAc–MeOH as the eluent to give 16 fractions. Fraction 6 (1.8 g) was separated on a silica gel column (230-400 mesh) into five subfractions (6a-6e). Subfraction 6a (50 mg) was applied to a reverse phase silica gel column (8 g) and eluted with methanol-water as eluent, to afford compound **1** (2 mg). Purification of subfraction 6b (40 mg) on a reverse phase silica gel (7 g) column with a gradient of methanol-water as eluent led to the isolation of compound **2** (3 mg). Subfraction 6c (70 mg) was purified on a reverse phase silica (12 g) column to give compound **3** (3.5 mg). 

*7-methoxy-3-propylidenephthalide (1)*

 Brown oil; ^1^H NMR (CDCl_3_ & CD_3_OD, 500 MHz) δ 7.62 (1H, t, *J* = 8.0 Hz, H-5), 7.21 (1H, d, J = 8.0 Hz, H-6), 6.93 (1H, d, *J* = 8.0 Hz, H-4), 5.63 (1H, d, *J* = 7.5 Hz, H-8), 4.01 (3H,s, -OCH_3_), 2.48 (2H, m, H-9), 1.14 (3H, t, *J* = 7.0 Hz, H-10), ^13^C NMR (125 MHz, CDCl_3_ & CD_3_OD): δ 158.7 (C-7), 147.0 (C-3), 142.3 (C-3a), 111.3 (C-4), 137.2 (C-5), 111.9 (C-6), 110.6 (C-7a(, 111.8 (C-8), 56.1 (-OCH_3_), 19.3 (C-9), 13.9 (C-10); HRESIMS (m/z = 205.071[M + H]^+^, calcd 205.086) for C_12_H_12_O_3_. 

*5-hydroxybutylidene phthalide (2)*

Brown oil; ^1^H NMR (CDCl_3_ & CD_3_OD, 500 MHz) δ 7.76 (1H, d, *J* = 8.0 Hz, H-7), 7.02 (1H, s), 6.97 (1H, d, *J* = 8.0 Hz, H-6), 5.57 (1H, t, *J* = 7.8 Hz, H-8), 2.43 (2H, m, H-9), 1.54 (2H, m, H-10), 0.98 (3H, t, *J* = 7.0 Hz H-11), ^13^C NMR (125 MHz, CDCl_3 _& CD_3_OD): δ 167.4 (C-1), 161.8 (C-5), 145.1 (C-3), 141.8 (C-3a), 127.3 (C-7), 118.2 (C-6), 117.0 (C-7a), 109.6 (C-8), 105.3 (C-4), 27.7 (C-9), 22.5 (C-10), 13.8 (C-11). 

*7-hydroxybutylidene phthalide (3)*

Brown oil; ^1^H NMR (CDCl_3_ & CD_3_OD, 500 MHz) δ 7.60 (1H, t, *J* =8.0 Hz, H-5), 7.30 (1H, d, *J* =8.0 Hz, H-6), 6.96 (1H, d, *J* =8.0 Hz, H-4), 5.84 (1H, d, *J* =7.7 Hz, H-8) 2.39 (2H, m, H-9), 1.65 (2H, m, H-10), 0.1.01 (3H, t,* J* =7.0 Hz, H-11), ^13^C NMR (125 MHz, CDCl_3 _& CD_3_OD): δ 158.3 (C-7), 147.7 (C-3), 142.6 (C-3a), 137.0 (C-5), 116.5 (C-6), 111.4 (C-4), 110.6 (C-7a), 109.7 (C-8), 28.0 (C-9), 22.8 (C-10), 13.7 (C-11). 

*Antibacterial activity*

*Bacterial strains*

*In-vitro* antibacterial activity of compounds was assessed against *Staphylococcus aureus* ATCC 25923, *Enterococcus faecium* (Vancomycin-resistant clinical strain) as Gram-positive bacteria and *Escherichia coli* ATCC 25922 and *Pseudomonas aeruginosa* PTCC1430 as Gram-negative bacteria. Some strains (ATCC strains and VRE) were kindly provided by Professor M.M. Feizabadi, Tehran University of Medical sciences. PTCC strain was purchased from Iranian Research Organization for Science and Technology.

*Determination of MIC*

Determination of the minimum inhibitory concentration (MIC) was carried out by the broth micro-dilution method as recommended by CLSI (Clinical Laboratory and Standard Institute) with minor modifications (10). The serial dilution of each compound was made in a range of concentrations of 256-0.015 μg/mL in sterile 96-well plates. The standard saline solution was prepared to get inoculant turbidity equal to 0.5 McFarland standards. The inoculants of the microbial strains were prepared from 20 h bacterial cultures that were adjusted to 0.5 McFarland standard turbidity and were further diluted (1:100) using MHB medium just before adding to the serially diluted samples. The plates were incubated for 24 h at 37 °C. MIC values were recorded as the lowest concentrations, which could inhibit the visible growth of the microorganisms. Each experiment was done in triplicate. Cefixime and chloramphenicol were used as the standard antibacterial agent.

## Result and Discussion

**Figure 1:**
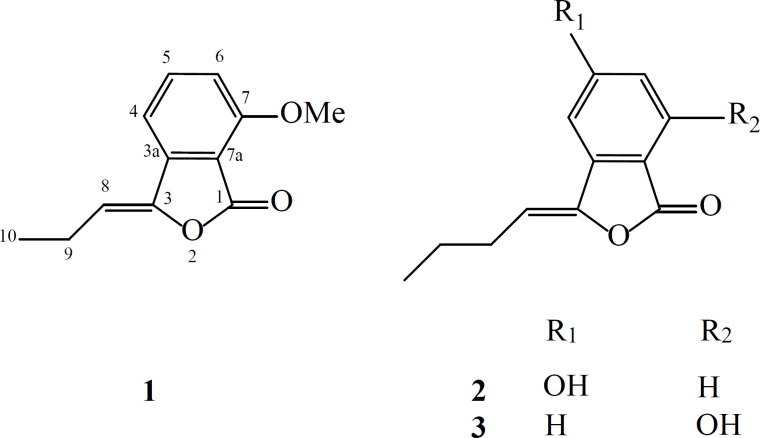
Chemical structures of the isolated compounds

Phytochemical analysis of the ethyl acetate extract from *L. officinale* led to isolation and identification of a new phthalide (**1**), together with two knowns compounds namely, 5-hydroxybutylidene phthalide (**2**) and 7-hydroxybutylidene phthalide (**3**) (Figure 1).

**Figure 2:**
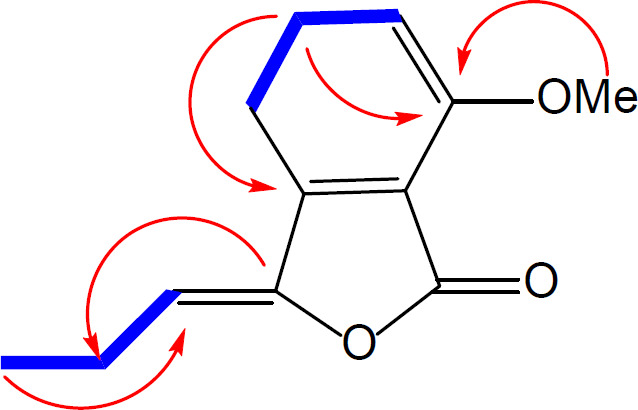
Selected HMBC () and COSY () correlations observed for compound **1**

Compound **1** was obtained as a brown oil and its molecular formula, C_12_H_12_O_3_ was established by HR-ESI-MS (m/z = 205.071[M + H]^+^, calcd 205.086) with 6-degrees of unsaturation. On the basis of APT and HSQC experiments, 12 resonances in the ^13^C NMR spectrum (Table 1) were resolved into two methyl (corresponding to one methoxy and one aliphatic methyl), one methylene, four methine and five quaternary carbons. The ^1^H NMR data (Table 1) confirmed the presence of a 1, 2, 3-trisubstituted aromatic ring [δ 6.93 (1H, d, J = 8.0 Hz), 7.20 (1H, d, J = 8.0 Hz), 7.62 (1H, t, J = 8.0)]. The NMR spectroscopic data of **1** showed high similarity to those of **3, **previously isolated from *Petroselinum crispum *[9], indicating that they are structurally related. Comparison of the ^1^H NMR and ^13^C NMR data of these two compounds indicated the loss of a CH_2_ group in side chain of compound **1**, which was also evidenced by the ^1^H-^1^H COSY and HMBC spectral data of compound **1** (Figure 2). 

Furthermore, an additional methyl signal was observed at δ_H_/δ_C_ 4.01/56.1, indicating the replacement of the phenolic hydroxyl group by a methoxy group. This was confirmed by a HMBC correlation from the methyl group to C-7. Thus, compound **1** was ascribed the name 7-methoxy-3-propylidenephthalide. 

The structure of the known compounds (**2** and **3**) was identified using ^1^H-NMR and ^13^C-NMR, as well as by comparison the data with those reported in literature (9). 

Apiaceae plants are rich source for phthalide compounds such as dimer and simple phthalides. Among simple phthalides, butylphthalides are the most abundant, but propylphthalides are rather rare (2, 3). So, presence of compound **1 **as propylphthalide in *L. officinale* is notable.

The activity of the isolated compounds was studied against strains of the Gram negative bacteria* Escherichia coli* and *Pseudomonas aeruginosa* and the Gram positive bacteria *Staphylococcus aureus* and vancomycin-resistant *Enterococcus *[VRE] *faecium*. Compound **3** showed activity against *S. aureus*, *E. coli*, and *E. faecium* with MIC values of 16, 64, and 128 μg/mL respectively, while compound **2** showed activity against only *S. aureus* and *E. coli* with MIC values of 128 and 256 μg/mL, respectively. Compound **1** showed reduced antibacterial activity against *S. aureus* and *E. coli* with MIC values of 256 μg/mL (Table 2). Thus, it appears that the free hydroxyl group is required for antibacterial activity in these derivatives.

Simple phthalide derivatives usually have low to moderate antibacterial activity with MIC values in the range of 4 – 0.128 mg/mL against *S. aureus* and *E. coli* (3). For example, ligustilide has previously been reported for its growth inhibitory activity against *E. coli *(MIC = 4 mg/mL), and *S*. *aureus* (1 mg/mL). Antibacterial activity was found for butylidenephthalide against *E. coli* and *S*. *aureus* with MIC values of 4 mg/mL, while (Z)‐ligustilide exhibited moderate antibacterial activity against *S. aureus *with MIC value of 1 mg/mL (3). 

**Table 1:** ^1^H and ^13^C NMR Spectral Data of Compound **1** (500 MHz for δ_H_; 125 MHz for δ_C_).

**Position**	**Compound 1**	
	**δ**_C _ **δ**_H_ ** (J in Hz)**	
1 3 3a 4 5 6 7 7a 8 9 10 OMe	Not observed 147.0 142.3 111.3 147.2 111.9 158.7 110.6 111.8 19.3 13.9 56.1	- - - 6.93 d (8.0) 7.62 t (8.0) 7.21 d (8.0) - - 5.63 t (7.5) 2.48 m 1.14 t (7.0) 4.01 s

**Table 2:** Antibacterial activity of phthalide derivatives **1-3**

**Compounds**	***Staphylococcus aureus***	***Enterococcus faecium*** ^*^	***Escherichia coli***	***Pseudomonas aeruginosa***
	MIC (μg/mL)			
1	256	>256	256	>256
2	128	256	128	>256
3	16	128	64	>256
Cefixime	2	16	4	64
Chloramphenicol	8	64	4	>256

^*^Vancomycin-resistant clinical strain

## Conclusion

In the present study, three phthalide derivatives were isolated from the roots of *L. officinale*, including new one. Their structures were elucidated by means of extensive 1D, 2D NMR spectroscopy. Also, the antibacterial activity of the compounds was studied against two Gram negative and two Gram positive bacteria strains. Among them, compound **3** hold a good potential for use in future studies due to their antibacterial properties. To our knowledge, this is the first report on the isolation and structure elucidation of the chemical constituents of *L. officinale *in Iran. All compounds are reported for the first time from *L. officinale*. Simple phthalides are not widely reported as showing any significant antibacterial activity. Therefore, the antibacterial activity of compound **3** is notable. 

## Supplementary material

1D and 2D NMR spectra of compound **1** can be found as Supporting Information.
